# Synchronizing Water and Nutrient Supply for Crop Production: Physiological Mechanisms, Technological Pathways, and the Case for Context-Specific Management

**DOI:** 10.3390/plants15213288

**Published:** 2026-10-25

**Authors:** Zhenyu Liu, Xicheng Hu, Wenbo Chen, Mingya Li, Yan Wang, Tingwu Liu, Qian Xu, Yuan Chen

**Affiliations:** 1 Jiangsu Key Laboratory for Eco-Agricultural Biotechnology Around Hongze Lake, Collaborative Innovation Center of Regional Modern Agriculture & Environmental Protection, School of Life Science, Huaiyin Normal University, Huai’an 223300, China; marmeislin@163.com (X.H.); cwb2002305402@163.com (W.C.); 19516891019@163.com (M.L.); yanwang@hytc.edu.cn (Y.W.); tingwuliu@163.com (T.L.); 2 School of Architecture and Planning and International Research Centre for Environmental Membrane Biology, Foshan University, Foshan 528000, China; xuqian@fosu.edu.cn; 3 Jiangsu Key Laboratory of Crop Genetics and Physiology, Co-Innovation Center for Modern Production Technology of Grain Crops, Yangzhou University, Yangzhou 225009, China

**Keywords:** water–fertilizer coupling, synchronized supply, root system architecture, aquaporins, rhizosphere processes, variable irrigation and fertilization

## Abstract

Synchronizing water and nutrient supply is central to high-yield, resource-efficient agriculture. We define synchronization operationally along three independently assessable dimensions: temporal overlap between supply and the crop demand window, spatial co-location of delivered water and nutrients with the zone of active roots, and matching of the nutrient-release rate to the crop uptake rate. Conventional simultaneous irrigation and fertilization does not necessarily ensure spatial and temporal matching between resource supply and crop demand. This review uses a four-part framework—costs of supply–demand mismatch, root sensing and response, regulatory technologies, and context-dependent variation—to trace the shift from simultaneous application to synchronized supply. Physiologically, drought restricts nutrient uptake via diffusion, mass flow, and active transport, while nutrient deficiency suppresses water-use efficiency through reduced photosynthesis and context-dependent changes in aquaporin regulation, root hydraulics, and hormonal signaling; combined stresses cause context-dependent oxidative and hormonal interactions. Roots integrate architectural plasticity, hydrotropic and nutrient-dependent foraging, hydraulic and chemical signaling, and rhizosphere processes to coordinate resource acquisition. Water–fertilizer technologies are grouped into four complementary technological pathways: fertigation, partial root-zone drying/alternate wetting and drying, sensor- and model-driven real-time control, and controlled-release fertilizer. Their effects diverge with soil physicochemical and hydraulic properties, genotype, seasonal climate, and spatial scale, so no universally optimal scheme is supported by current evidence. Key bottlenecks are weak in situ root–soil observation, lack of coupled multi-process models, and scarce field validation. Future progress requires high-throughput root phenotyping, AI prediction, low-cost sensing, and multi-objective optimization for context-specific management.

## 1. Introduction

Global agriculture faces a dual bind: water scarcity coexists with the over-application of fertilizer. Irrigation accounts for roughly 70% of global freshwater withdrawals, a withdrawal share rather than the share of consumptive water use, while excessive nitrogen inputs not only push use efficiency steadily downward but also drive nitrate contamination of groundwater and greenhouse-gas emissions [1]. Against this background, water–fertilizer coupling has come to be seen as a core strategy for raising yields and resource-use efficiency at the same time [2]. Radmehr and Brorsen [3] estimated that adjusting nitrogen application on US maize and wheat to socially optimal levels, 20–37% below current rates, could yield net social benefits of up to US$16 billion per year.

Conventional water and fertilizer management relies on simultaneous application, in which irrigation and fertilization are carried out as one operation: urea broadcast during furrow or flood irrigation, or a concentrated fertilizer solution injected in a single pass through a drip system. The implicit assumption is that physical co-delivery to the root zone is enough for the crop to take up both and gain the benefit of synergy. Field evidence, however, shows that simultaneity is not synchronization. Crop demand for water and for nutrients follows different temporal curves, and the soil’s capacity to supply each depends on texture, temperature, and microbial activity [4]. As a result, simultaneous water–fertilizer combinations frequently fall into mismatch—water arrives before the nutrients, or fertilizer is applied with too little water—and a substantial fraction of irrigation water and fertilizer is lost to deep percolation, ammonia volatilization, or denitrification before it ever enters the crop. A global assessment of sugarcane systems found nitrogen rate and crop-residue management to be the dominant controls on ammonia volatilization and emissions [5].

The concept of synchronized supply is a direct response to this mismatch. Its core meaning is straightforward: match the effective supply of water and nutrients to the crop’s real-time demand at each growth stage and to the soil’s real-time supply capacity, precisely and along both spatial and temporal dimensions. This is not a repackaging of drip fertigation or other existing technologies. It begins with the degree of coupling between the crop’s demand curve and the soil’s supply capacity curve, and it seeks, through technical means, to keep shrinking the gap between the supply window and the demand window.

We define synchronized water–nutrient supply as the targeted matching of the spatiotemporal dynamics of supply to crop demand along three independently assessable dimensions: (i) temporal synchronization, the overlap between the water and nutrient supply window and the crop demand window at each growth stage; (ii) spatial synchronization, the co-location of delivered water and nutrients with the zone of active roots; and (iii) rate synchronization, the matching of the nutrient-release or delivery rate to the crop uptake rate. Synchronization is a property of the supply–demand matching structure, assessed before outcomes are measured; it is therefore not equivalent to any observed gain in WUE, NUE, or yield, which are consequences rather than criteria. Loss minimization is an outcome used to verify synchronization, not part of its definition. A management practice qualifies as synchronization only when it reduces the supply–demand mismatch along at least one dimension at constant or reduced total input and when that reduction is attributable to improved matching rather than to a change in the level of supply. Efficiency gains obtained simply by raising applied water or fertilizer are therefore excluded, whereas gains obtained by shifting the supply window toward the demand window, relocating supply into the root zone, or aligning the release curve with the uptake curve are included. Synchronization and synergy are distinct concepts: a synchronized strategy can raise resource-use efficiency without producing a statistically or physiologically synergistic water × nutrient interaction, and joint management that improves yield should not automatically be read as physiological synergy.

The degree to which water and fertilizer management has been synchronized varies widely across the world. Here, NUE denotes nitrogen partial factor productivity (PFP_N_), defined as crop yield per unit of applied nitrogen. Because the numerical values previously used to contrast production systems cannot be verified here with a common reference condition and should not be compared with nitrogen recovery, agronomic, or physiological efficiency, they have been removed. Differences in technology adoption and nutrient management among production systems nevertheless underscore the need for low-barrier, robust synchronization schemes.

To assess the scientific value of synchronized supply, this review builds a four-part, progressively nested framework: the cost of supply–demand mismatch, the mechanisms of sensing and response, the technological pathways, and the sources of effect divergence. The physiological cost of mismatch establishes why synchronization is necessary; root sensing and response establish that it is physiologically feasible; the four complementary technological pathways identify the distinct means by which it can be pursued; and the patterns of divergence expose the limits of any universal scheme. Throughout, conventional uniform irrigation and fertilization (simultaneous application) serves as the reference, and we assess outcomes along four dimensions: water-use efficiency (WUE), NUE, yield, and environmental impact [6,7]. In terms of coverage, we prioritize high-impact work from the past two decades (2006–2026), alongside earlier foundational studies and the latest advances, so as to present the full arc from empirical simultaneity to mechanistic synchronization.

A note on evidence hierarchy is warranted. This review integrates findings from molecular studies, split-root experiments, greenhouse trials, field experiments, simulation modeling, meta-analyses, and remote-sensing studies. These evidence types carry different inferential weight: mechanistic conclusions established under controlled split-root systems should not be automatically extrapolated to field-scale yield responses, and simulation outputs are not equivalent to experimental validation. Throughout the review, we endeavor to specify the nature and strength of the evidence supporting each major claim. Because water- and nitrogen-use efficiency are used at different scales and with different definitions, we specify the metric whenever numerical values are reported: water-use efficiency as intrinsic (photosynthesis relative to stomatal conductance), whole-plant, crop water productivity (biomass or yield per unit evapotranspiration), or irrigation water productivity; and nitrogen-use efficiency as recovery efficiency, agronomic efficiency, physiological efficiency, or partial factor productivity. The scale-transition problem, from pot to plot to farm to region, is treated as a cross-cutting theme rather than a concern of a single section.

We searched Web of Science, Scopus, and Google Scholar using the following keyword combinations: (“water–fertilizer coupling” OR “fertigation” OR “water–nitrogen interaction” OR “synchronized supply” OR “nutrient-use efficiency”) AND (“root system architecture” OR “aquaporin” OR “rhizosphere” OR “partial root-zone drying” OR “controlled-release fertilizer” OR “precision irrigation”). The search was restricted to English-language peer-reviewed articles published between January 2006 and June 2026. After removing duplicates (*n* = 847), the initial pool of approximately 2500 records was screened by title and abstract relevance to water–nutrient synchronization, from which approximately 410 full-text articles were assessed. The final body of the cited literature comprises 118 sources prioritized for methodological rigor, quantitative evidence, and representativeness across crops and environments.

## 2. Physiological Costs of Water–Fertilizer Supply–Demand Mismatch

Mismatch between water and fertilizer supply and crop demand does more than waste resources in obvious ways, as surplus irrigation water percolates into deeper soil and excess nitrogen escapes as gas or ions. Its deeper cost lies in a cascade of physiological damage. These injuries run through a closed loop—drought, impaired nutrient uptake, constrained photosynthesis, declining WUE, oxidative damage, and hormonal imbalance—that amounts to a hidden yield penalty [8]. Direct quantification of this penalty is scarce: we are not aware of field experiments that deliberately impose misaligned water and nutrient supply against matched aligned controls, so the magnitude of the yield loss under mismatch is inferred from the mechanistic and stress-interaction studies reviewed below rather than measured under field conditions. We treat this as an open research gap and return to it in Section 6. This section dissects that cascade along three dimensions: the barriers to nutrient uptake under drought, the reciprocal inhibition of WUE by nutrient deficiency, and the interaction between oxidative damage and hormonal disturbance.

### 2.1. The Multiple Barriers to Nutrient Uptake Under Drought

Soil water deficit restricts nutrient acquisition through processes that operate at two distinct levels: soil-level transport of nutrients to the root surface, comprising diffusion and transpiration-driven mass flow, and membrane-level uptake across the root-cell membrane, which depends on root metabolic activity and active transport. These describe different physical and physiological scales and are treated separately below, rather than as equivalent “uptake routes”. Water deficit slows diffusion, reduces mass flow, and suppresses root metabolic activity, and the sensitivity of nitrogen (N), phosphorus (P), and potassium (K) to each process is element-specific.

Along the diffusion route, declining soil water content shrinks the volume of the soil solution and lengthens the tortuous path of ions, sharply lowering the effective diffusion coefficient of N (mainly nitrate, NO^3−^) and of ammonium from the bulk soil to the root surface. Marschner et al. [9] pointed out that rhizosphere microorganisms and roots compete intensely for iron and phosphorus; under drought, microbial competitiveness falls as carbon supply declines, and the microbial mobilization of nutrients weakens as well. Along the transpiration-driven mass-flow route, drought-induced stomatal closure lowers the leaf’s transpiration pull and thus weakens convective mass transport of ions to the root surface. Along the root-activity route, water deficit inhibits root respiration and lowers plasma membrane H^+^-ATPase activity, weakening the proton-gradient-dependent active uptake of ions. Working in maize, Guo et al. [10] documented this suppression directly. Heslot et al. [11] developed a genomic-selection framework that integrates environmental covariates with crop models, providing a methodological basis for predicting genotype × environment interactions. Combined heat and drought reshaped root morphological and physiological traits, cutting root nitrogen-uptake efficiency by 9.0% and 10.4% under the individual stresses but by 18.0% under the combined stress, with coordinated inhibition of the key nitrogen-assimilation enzymes NR, GS, and GOGAT.

At the molecular level, drought down-regulates root nitrate transporters (NRT) and ammonium transporters (AMT), limiting nutrient uptake across the membrane at its source. In several crops, the high-affinity NRT2 genes fall sharply under drought, greatly weakening the root’s ability to absorb nitrate at low concentrations, and AMT1 expression is likewise suppressed by water deficit [12]. This molecular suppression deepens the mismatch between supply and uptake at the whole-plant scale.

Notably, these processes respond to water deficit on different timescales. Diffusion limitation appears first, once soil water content falls to about 60–70% of field capacity; mass-flow limitation follows the decline in transpiration; and reduced root physiological activity typically emerges after about 5–7 days of sustained drought. These thresholds are indicative ranges observed under the specific crop, soil, and experimental conditions noted in Table 1, not universally applicable values. This sequence means that even when total soil nutrients are adequate, a short drought can induce a physiological nutrient deficiency [13]. In winter wheat, Fu et al. [14] further showed that combined drought and salinity sharply reduced root hydraulic conductivity (Lpr), root length, and root surface area, with N and K declining in both roots and shoots, evidence that drought restricts nutrient uptake through both physical shrinkage and physiological suppression. In wheat under combined microplastic and water-deficit stress, Li et al. [15] found that an acquisitive functional-trait strategy alleviated growth inhibition more effectively than a conservative one.

### 2.2. Reciprocal Inhibition of Water-Use Efficiency by Nutrient Deficiency

The cost of mismatch is not one-directional, as though water shortage simply limited nutrient uptake. Nutrient deficiency in turn weakens WUE, forming a second damage pathway in which a lack of fertilizer lowers water-use efficiency.

Throughout this section, water-use efficiency refers to leaf-level instantaneous WUE and whole-plant WUE unless otherwise stated; these are distinct from intrinsic WUE (photosynthesis relative to stomatal conductance), from crop water productivity based on biomass or yield per unit evapotranspiration, and from irrigation water productivity, and the terms are not interchangeable. Nitrogen deficiency suppresses WUE through two mechanisms. It reduces the content and activity of Rubisco, limiting photosynthetic carbon assimilation so that carbon fixed per unit of transpired water falls; that is, instantaneous WUE declines. It also weakens stomatal sensitivity to the leaf-to-air vapor pressure deficit (VPD), so transpiration cannot be reduced effectively under high VPD, increasing non-productive water loss [16]. Phosphorus deficiency has a more hydraulic character: in the species and isoforms examined so far, under P deficiency the expression of root aquaporin (AQP) genes, especially the plasma membrane intrinsic protein (PIP) subfamily, falls markedly, and root hydraulic conductivity declines by about 30–50%; aquaporin abundance, gating, localization, and isoform-specific expression vary with nutrient, tissue, species, stress severity, and duration, so this response is context-dependent rather than uniform [17]. In Leymus chinensis, Li et al. [17] confirmed that the decline in AQP expression and in root hydraulic conductivity occurred together under P deficiency, while the Casparian strip and suberin lamellae in the root endodermis thickened, further restricting radial water transport. The effect is essentially a physiological drought: even with ample soil water, reduced root water transport leaves the leaf facing water deficit.

In rice, Ishikawa-Sakurai et al. [18] showed that nitrogen availability affects root hydraulic conductivity by regulating AQP expression. Nitrogen starvation down-regulated root-specific PIP genes and lowered root hydraulic conductance, and both recovered quickly once nitrogen was restored. In Arabidopsis, Li et al. [19] resolved the layered mechanism: nitrate signaling regulates AQP function through an NRT2.1-dependent pathway, rather than through nitrogen metabolites themselves. Nitrogen thus influences water-use efficiency not only indirectly through photosynthesis but also directly by setting the capacity of the root’s water transport channels.

Kurepa and Smalle [16] traced the molecular basis of this reciprocal inhibition to hormonal hierarchy: auxin promotes root growth and AQP expression, whereas cytokinin promotes shoot growth and suppresses root expansion. Under combined drought and nitrogen deficiency, auxin–cytokinin antagonism intensifies, and the resulting root-to-shoot imbalance further widens the water supply–demand gap. In short, nutrient deficiency lowers not only the yield gained per unit of nutrient but also, through AQP down-regulation and hormonal imbalance, the yield gained per unit of water, offsetting the efficiency gains of water-saving irrigation [4].

### 2.3. Combined Water and Nutrient Stress: Oxidative Damage and Hormonal Disturbance

When water deficit and nutrient deficiency occur together, their effects can be additive, synergistic, antagonistic, or emergent, depending on stress intensity, sequence, developmental stage, genotype, and environmental context. In the studies reviewed here, the reactive oxygen species (ROS) and hormonal responses show non-linear interactions rather than simple addition.

At the ROS level, the rise in NADPH oxidase activity induced by drought combines with insufficient substrate for antioxidant enzymes (SOD, CAT, APX) caused by nitrogen deficiency to produce a production–scavenging imbalance: ROS are generated faster than they can be removed, and hydrogen peroxide and superoxide accumulate in root tips and leaves. In maize, root oxidative damage under combined heat and drought exceeded that under either stress alone, root respiration was suppressed most severely, and the resulting cellular energy deficit further limited nitrogen assimilation [10]. In cotton, drought altered root morphology, raising the proportion of fine roots and elongating root hairs, and accelerated fine-root turnover [20]. The strategy of letting fine roots die faster while producing new ones increases absorptive area in the short term, but it depends on an adequate carbohydrate supply, precisely what nitrogen deficiency curtails, undermining its sustainability.

At the hormonal level, drought-induced ABA accumulation and ROS form a positive-feedback loop [21]: ABA activates RBOH (respiratory burst oxidase homolog) to promote ROS production, and ROS in turn stimulate ABA synthesis by activating NCED (9-cis-epoxycarotenoid dioxygenase), keeping stomata excessively closed for long periods.

At the same time, the lower cytokinin levels under nitrogen deficiency restrict cell division and leaf expansion, adding to the conservative regulation imposed by ABA. Drought also down-regulates auxin biosynthesis genes and up-regulates jasmonate synthesis genes, altering the root’s hormonal balance [13]. At the organ scale, this dysregulation shows up as roots entering maintenance mode prematurely as carbon allocation shifts belowground, and as shoots suffering insufficient photosynthetic area from excessive stomatal closure and restricted leaf expansion [22]. The net effect under the conditions studied is that cumulative yield loss under water–fertilizer mismatch can exceed the simple sum of losses from insufficient irrigation efficiency and insufficient fertilization efficiency, and this hidden yield penalty is often masked by conventional yield-increase metrics. Whether, and by how much, the whole exceeds the sum of the parts depends on the same contextual factors and still requires direct field quantification with deliberately misaligned versus aligned water and nutrient supply. This is the most fundamental physiological reason for moving water and fertilizer management from simultaneous application toward synchronized supply.

**Figure 1:**
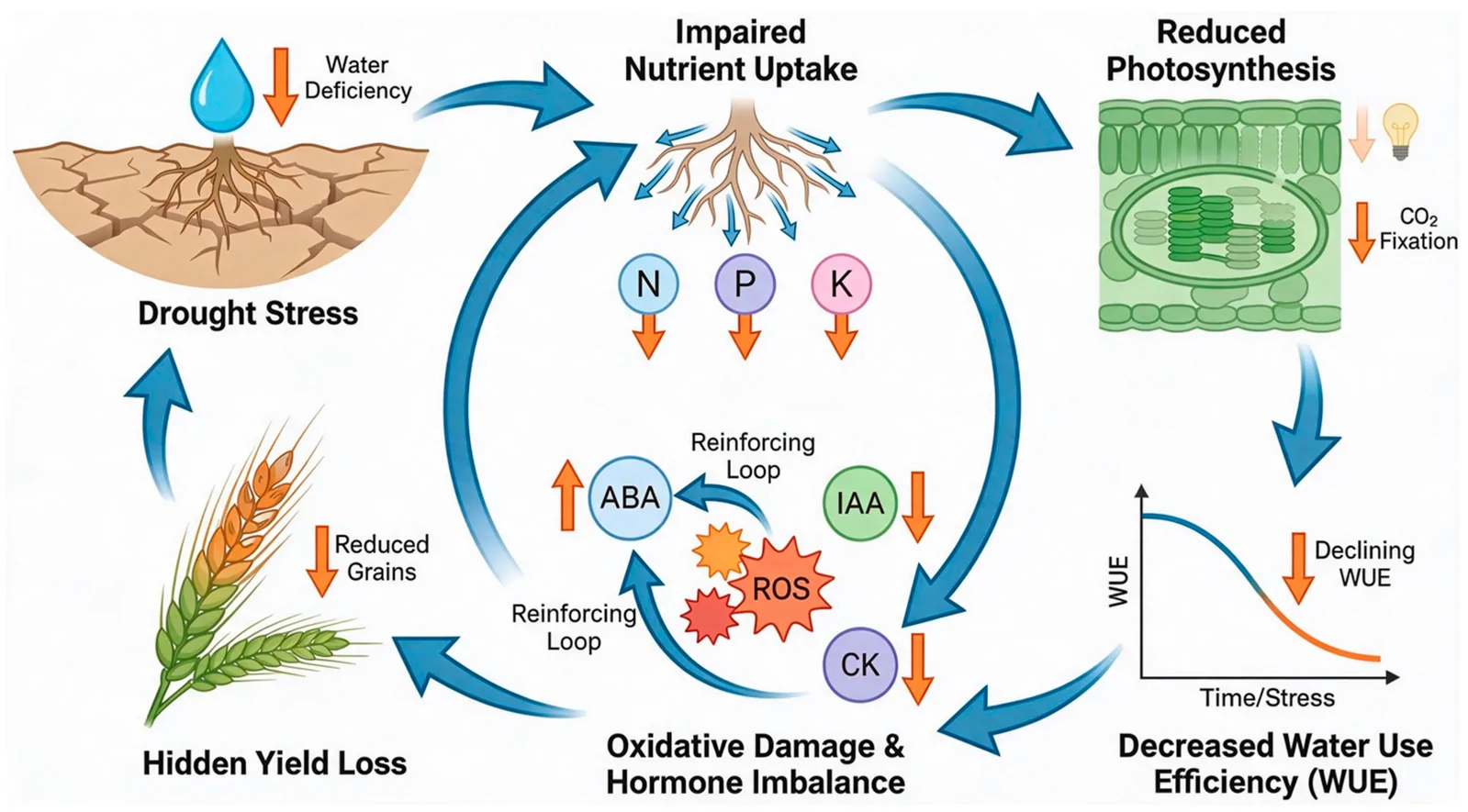
Schematic of the cascading physiological costs of water–fertilizer supply–demand mismatch.

As Figure 1 shows, drought stress, impaired nutrient uptake, declining photosynthesis, lower WUE, oxidative damage, and hormonal disturbance form a mutually reinforcing cascade. The defining feature of this system is the interdependence of its parts: improving any single link, adding irrigation water, for instance, may fail to raise yield because other links remain limiting. This explains why the efficiency of simultaneous water–fertilizer management is hard to improve substantially.

Table 1 summarizes the soil-level and membrane-level processes in terms of mechanism, affected nutrients, response timing, and severity. The two soil-level processes, diffusion and mass flow, respond faster to changes in water, whereas the membrane-level process, which depends on enzyme synthesis and energy metabolism, has the longest recovery lag. After irrigation restores soil water content, the root’s active-uptake capacity may take days to recover, but the recovery kinetics depend on species, nutrient, drought intensity and duration, root damage, transporter regulation, metabolic status, and rewatering conditions, so this is a context-dependent tendency rather than an intrinsic universal limit. The lag is nonetheless a constraint that simultaneous application cannot correct by simply increasing irrigation frequency.

**Table 1:** Nutrient-acquisition limitation under drought across two levels: soil-level transport to the root surface and membrane-level uptake.

Level	Process	Main Mechanism	Nutrients Affected	Response Time	Key References
Soil-level transport to the root surface	Diffusion	Lower diffusion coefficient	N (NO_3_^−^), P	Earliest (about 60–70% of field capacity; observed in maize [10])	[10]
Soil-level transport to the root surface	Mass flow	Weaker transpiration pull	N, K	Intermediate (with stomatal closure)	[13]
Membrane-level uptake at the root surface	Active uptake	Lower H^+^-ATPase activity	N, P, K	Latest (about 5–7 days of drought; indicative range, context-dependent)	[10,22]

Note: The response sequence and the nutrients affected are based on the combined-stress maize experiment of Guo et al. [10] and the hormone–nutrient interaction review of Dodd et al. [13]. The thresholds given (60–70% of field capacity; 5–7 days) are indicative ranges reported for particular crops, soils, and experimental conditions and should not be read as universal constants; the cited studies are controlled and glasshouse experiments rather than multi-environment field validation. The delayed recovery of the active-uptake route also involves the cascade of root carbon-energy deficit and nitrogen-assimilation inhibition under drought described by Kang et al. [22].

## 3. Root Sensing and Response to the Spatiotemporal Pattern of Water and Nutrients

The root is the pivotal interface linking soil water and nutrient supply to aboveground productivity. Under natural conditions, soil water and nutrients are highly heterogeneous in space and time: phosphorus accumulates in the topsoil, water is stored at depth, and nitrogen redistributes down the profile with irrigation and rainfall [23]. The evidence reviewed here is uneven across nutrients: most mechanistic and technological studies concern nitrogen, whereas phosphorus and potassium differ from nitrate in soil mobility, transport to the root, rhizosphere chemistry, and response to soil water content, so conclusions should be read as water × nitrogen unless a nutrient is named explicitly. Here we propose a three-component conceptual framework in which roots convert this physical pattern into a coordinated configuration of bioavailable resources, as the physiological basis of synchronized water and fertilizer supply.

### 3.1. Root Architectural Plasticity and Spatial Orientation

The plasticity of root system architecture (RSA) is the plant’s primary spatial strategy for coping with heterogeneous soil resources [24,25]. In soil with heterogeneous water and nutrients, RSA optimizes foraging along three axes: the direction of primary-root elongation, navigating through tropic growth; lateral-root density and branching angle, proliferating locally in response to resource patches; and root-hair development, enlarging absorptive surface area. Karlova et al. [25] reviewed the molecular networks governing RSA plasticity under five abiotic stresses (heat, cold, drought, salinity, and waterlogging) and showed that adaptive root responses involve complex crosstalk among auxin, ABA, ethylene, and cytokinin.

Hydrotropism and nutrient-dependent local foraging are the two core spatial-orientation mechanisms. Only hydrotropism and a limited set of tropic responses are demonstrated as directional growth; the root’s response to nutrient patches is better described as local architectural plasticity and foraging rather than chemotropism in the strict sense. Dietrich et al. [26] uncovered the distinctive cellular basis of hydrotropism: unlike gravitropism, which is sensed in the root cap, hydrotropic sensing occurs in cortex cells of the elongation zone and depends on the ABA-signaling kinase SnRK2.2 and the hydrotropism-specific factor MIZ1. Laser ablation showed that roots still respond hydrotropically after the root cap and meristem are removed, indicating a differential-growth pathway independent of gravitropism. Uzilday et al. [27] integrated the roles of ABA, ROS, and calcium signaling in hydrotropism, suggesting that after a water gradient is sensed, asymmetric ABA distribution or calcium signaling induces differential cell elongation.

For nutrient-patch responses, nitrate is not only a nitrogen source but a key signal regulating RSA. In Arabidopsis, the high-affinity nitrate transporter NRT1.1 (CHL1/AtNPF6.3) senses external nitrate at the root tip and, by regulating the expression and subcellular localization of the auxin efflux carrier PIN, alters the auxin gradient in the root tip and thereby controls lateral-root primordium initiation and elongation [28]. Nitrate therefore acts through both a local sensing pathway and a systemic nutritional pathway, and root proliferation toward nitrate-rich zones can reflect altered nitrogen status and carbon–nitrogen balance as much as directional sensing. Giehl et al. [29] emphasized the broad element specificity of RSA responses: a local nitrate-rich zone promotes lateral-root elongation rather than increased density, a local phosphate-rich zone increases lateral-root density and root-hair length, and local ammonium enrichment stimulates lateral-root branching, responses that are local architectural adjustments rather than demonstrated directional tropic growth. In a review, Huang and Zhang [30] synthesized the molecular regulation of RSA plasticity by external phosphate, showing the coordinated involvement of actin filaments, post-translational modification, and auxin, ethylene, and cytokinin signaling. This element-specific plasticity lets roots make integrated three-dimensional architectural decisions within combined water and nutrient gradients.

Different RSA strategies carry different energy costs. Lateral-root proliferation and root-hair development are high-return, high-cost local investments, whereas deep primary-root growth is a low-return, low-cost broad search. In dry and semi-arid soils with pronounced vertical gradients of water and nutrients, a deep-root strategy may be more adaptive than shallow proliferation [31]. Esfahani and Sonnewald [23] proposed ideotype models for maize and common bean—topsoil-foraging, subsoil-foraging, and dual-layer types—each with a different efficiency in allocating water and phosphorus. This architecture–cost trade-off bears directly on the design of synchronized supply: promoting deep roots in sandy soil may matter more than promoting shallow laterals in clay, so the technical approach should follow soil and crop type. Colombi et al. [32] distinguished plasticity from elasticity in an energy-budget framework, arguing that reversible responses that recover after stress is relieved, that is, elasticity, carry a lower carbon cost, whereas sustained, irreversible change, that is, plasticity, can accumulate excessive carbon cost. This does not mean that irreversible structural adjustments lack adaptive value: such developmental changes can support longer-term adaptation, so the distinction is better read as one between short-term plastic responses and longer-term developmental adaptation, each with its own cost–benefit balance. This framework reframes the cost–benefit balance of RSA strategies.

### 3.2. The Dual-Channel Hydraulic and Chemical Signaling System

After sensing heterogeneity in soil water and nutrients, roots transmit information to the shoot along two channels, hydraulic and chemical, to coordinate root and canopy.

The hydraulic signal is a fast physical signal. When soil water content declines in part of the root zone, root-cell water potential falls, and tension is relayed through the continuous xylem water column to the leaves within minutes to tens of minutes, causing rapid declines in stomatal conductance and transient reductions in leaf expansion. The speed depends on xylem vessel diameter and connectivity, and in large-vessel species the transmission velocity is 0.5–1.0 mm s^−1^, with the tension signal reaching the leaves within minutes to tens of minutes, constituting a minute-scale rapid-response channel [33].

The chemical signal is a slower hormonal signal. When the root zone is heterogeneous in water, as under PRD, roots on the dry side synthesize and export ABA into the xylem transpiration stream, which carries it to the guard cells and induces stomatal closure. Using a split-root grafting system, Dodd et al. [34] quantified how roots in different water states contribute to the total transpiration stream and to xylem ABA concentration: once the dry-side soil water fell below a threshold, the dry roots’ share of the transpiration stream declined, and their ABA export fell with it; but when alternate irrigation rewetted the dry side, ABA stored in the roots was released into the transpiration stream in a pulse, producing a one-off high-concentration ABA signal. This revealed the key role of alternation frequency in the PRD effect. More recently, in soybean, Puértolas et al. [35] confirmed that under PRD the hormonal outputs of the dry- and wet-side roots are independent: the dry side exported mainly ABA and the wet side mainly jasmonic acid (JA), while ACC, the ethylene precursor, increased on both sides. More importantly, split-root grafting showed that this differential output did not depend on hormone recirculation from shoot to root but was controlled directly by the local water status of the roots.

The complementary timescales of hydraulic and chemical signaling are the core advantage of the root signaling system. The hydraulic channel provides an immediate-response route, acting within minutes, that lets the plant adjust stomata when transpiration demand suddenly rises, while the chemical channel provides a steady-state regulation route, acting over hours to days, that lets the plant adjust growth strategy to sustained soil water conditions [12]. Together they form a temporally tiered regulatory network in which a fast channel prevents acute dehydration damage and a slow channel secures long-term resource-use efficiency. This is the physiological basis for the paradoxical water-saving-without-yield-loss gains of PRD and AWD. Comparative studies of isohydric and anisohydric grape cultivars show that the weight of this dual-channel system differs markedly among genotypes [36], which means that optimizing synchronized supply has to match a cultivar’s signal preference.

### 3.3. Rhizosphere Microdomain Processes and Coupled Water–Nutrient Activation

The rhizosphere, the roughly 0.5–4 mm shell of soil influenced by root activity, is the first reactor in which soil water–nutrient heterogeneity is converted into bioavailability [37]. Within it, root exudates, microbial communities, and soil physicochemical processes form a complex interaction network that turns a physically heterogeneous distribution of water and nutrients into a temporally coordinated bioavailable supply.

Root exudates are the plant’s central means of actively regulating the rhizosphere environment. They include low-molecular-weight organic acids (citrate, malate, oxalate), amino acids, sugars, and secondary metabolites [38]. In a review, Chai and Schachtman [39] summarized the functions of root exudates under drought, aluminum toxicity, and nitrogen, phosphorus, and iron deficiency, noting that specific exudates such as malate and citrate not only mobilize nutrients directly through chelation but also act as signals that recruit beneficial microorganisms, indirectly enhancing resource-use efficiency. Under P deficiency, Pollet et al. [40] found that white lupin produced more root biomass and carboxylate exudates at intermediate P supply, and that even very low exudate concentrations, below 100 ppb, promoted mineral dissolution across several soil layers. By exuding large amounts of citrate, white lupin and similar species lower rhizosphere pH by 1–2 units while chelating iron and aluminum, releasing phosphate fixed by iron and aluminum oxides [41]. Under iron deficiency, grasses secrete mugineic-acid-type phytosiderophores that chelate iron with high affinity and transport it into the root. Root exudates such as oxalate and glucose can also release adsorbed organic matter and nutrients from mineral surfaces directly through chelation, and indirectly accelerate the decomposition of mineral-bound organic matter by stimulating microbial extracellular enzyme activity; this dual-pathway mobilization markedly strengthens rhizosphere nutrient supply. Mumtaz et al. [42] described the rhizosphere as a tightly coupled physicochemical–biological system in which microbial metabolism, root exudates, and mineral weathering jointly regulate nutrient flux and bioavailability through feedback loops.

Exudates also regulate nutrient availability indirectly by shaping the rhizosphere microbiome. Zhalnina et al. [43] showed a precise coupling between the temporal chemistry of root exudates and microbial substrate preferences: bacteria in the oat rhizosphere preferentially consumed aromatic organic acids such as nicotinic, shikimic, salicylic, cinnamic, and indole-3-acetic acid, and this preference aligned closely with the developmental shifts in exudate composition, a form of metabolic coupling. Beattie [44] called this coupling metabolic coupling on roots, noting that plants selectively enrich beneficial microbial taxa by finely tuning the secretion of specific metabolites. This offers a basis for deliberately reengineering rhizosphere communities by manipulating exudate composition. Using P isotope dilution, Spohn et al. showed directly that simulated root exudates such as glucose and alanine raised the gross mineralization rate of microbial organic P by 20–31-fold within a short time, with the effect depending on the exudate, implying that plants can finely control the pace of rhizosphere P supply by changing exudate composition. Using ^33^P isotope dilution, Spohn et al. [45] showed directly that simulated root exudates such as glucose and alanine raised the gross mineralization rate of microbial organic P by 20–31-fold within a short time, with the effect depending on the exudate, implying that plants can finely control the pace of rhizosphere P supply by changing exudate composition.

Drying and rewetting (DRW) is another key process in the rhizosphere. Dodd et al. [13] noted that soil drying alters root hormone synthesis by limiting root water uptake, while rewetting triggers two effects: a physical effect, in which slaking of soil aggregates releases physically protected organic matter and nutrients, and a biological effect, in which partial microbial death during drought is followed by rapid mineralization after rewetting, the so-called Birch effect, a pulse of nutrient release. Wang et al. [46] further reported that the frequent DRW cycles induced by PRD enhanced soil nutrient mineralization and raised the bioavailability of N and P, though uptake efficiency depended on competition between plants and microorganisms. Together, these rhizosphere processes—in which microorganisms may enhance nutrient availability, compete with roots for nutrients, or immobilize them depending on context—show that synchronized water and fertilizer supply should not focus only on the operational coupling of irrigation and fertilization. It should also harness the intrinsic biophysical coupling of the rhizosphere, regulating exudate composition and irrigation regime to activate microbial nutrient mobilization and water retention, and so achieving a two-layer coordination of operational and biological synchronization.

Water × phosphorus synchronization differs from water × nitrate synchronization. Because phosphate is largely immobile in soil and reaches roots mainly by diffusion, its acquisition depends less on temporal matching of water and nutrient supply and more on spatial co-location with active roots, on root-hair and mycorrhizal exploration, and on rhizosphere modification of pH, organic-acid exudation, and phosphatase activity, together with soil sorption. Synchronized management for phosphorus therefore emphasizes spatial synchronization and rhizosphere activation, whereas for nitrate it emphasizes temporal matching and control of leaching and gaseous loss.

**Figure 2:**
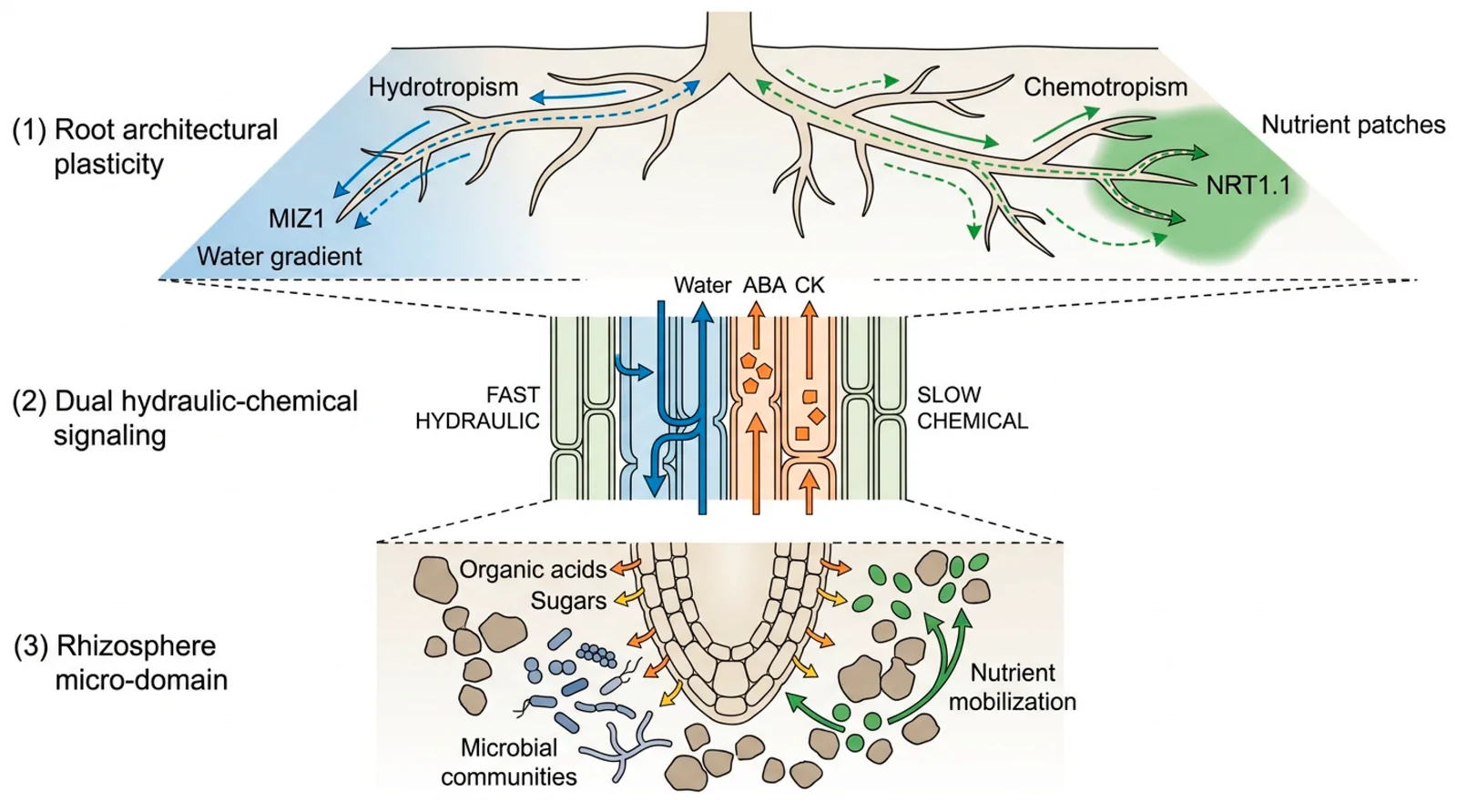
The root as the hub of synchronized water and fertilizer supply: a three-tier sensing–response system.

As Figure 2 shows, roots form a complete sensing–response system through three tiers that act together: architectural plasticity, dual-channel hydraulic and chemical signaling, and rhizosphere regulation. Its defining feature is the coupling of information flow across tiers. Spatial rearrangement at the architectural level provides the physical basis for signaling, while chemical modification in the rhizosphere feeds back on architectural decisions, giving rise to a complex adaptive system that goes beyond a simple stimulus–response model.

## 4. From Simultaneous to Synchronized: The Evolution of Regulatory Pathways

The evolution of water–fertilizer technologies from simple simultaneous application to precise synchronized supply can be read as an iterative narrowing of the gap between the supply window and the demand window. We organize these technologies not as a historical sequence but as four complementary technological pathways that differ in the level at which they intervene and in the synchronization dimension they address (Table 2): fertigation, which acts on resource delivery; partial-zone and alternate irrigation, which manipulates root-zone water heterogeneity and plant signaling; sensor- and model-driven real-time control, which operates at the decision and control level; and controlled-release fertilizer, which regulates nutrient-release kinetics. These pathways are complementary and partly independent, and one is not necessarily a later evolutionary stage of another; controlled-release fertilizer, for example, is not intrinsically more advanced than sensor-based control. Comparing effect sizes across the pathways also requires caution, because the reference or control treatments, crop species, experimental duration, sample size, and research scale differ among the underlying studies, so percentage improvements are not directly comparable.

As Table 2 shows, the pathways differ in intervention level, synchronization dimension, and core mechanism, and they do not replace one another: under a given combination of soil, genotype, and climate, combining technologies from different pathways may provide complementary benefits relative to a single approach, although direct comparative evidence remains limited. In the North China Plain, Zhang et al. [55] used UAV data to achieve dynamic variable-rate fertigation in a center-pivot system, saving 31% of water while raising yield by 13%. In a systematic review of 266 studies, Singh and Singh [51] reported that precision fertigation integrating IoT sensors, TDR soil moisture monitoring, and GreenSeeker canopy spectroscopy raised WUE by 61–64.1% and yield by 12–35%, giving a macro reference for the real benefits of the four technical pathways.

### 4.1. Fertigation: From Simple Mixing to Initial Spatial Synchronization

Fertigation, the first synchronization pathway, made its central contribution by achieving an initial spatial synchronization of water and fertilizer supply: it compressed the delivery space from the whole field to the root zone. Drip fertigation is the archetype. Injecting liquid fertilizer into the irrigation water and delivering it precisely to the crop root zone through emitters reduces deep percolation and soil evaporation losses while lowering the leaching risk of nitrate nitrogen.

Li et al. [7] quantified the benefits of drip fertigation over conventional flood irrigation with broadcast fertilizer in a China-wide meta-analysis: drip fertigation significantly raised yield by 12.0%, water productivity by 26.4%, and NUE by 34.3% while cutting crop evapotranspiration by 11.3%. Effects differed by crop, with potato showing the largest yield gain (40.3%) and fruit the largest NUE gain (68.2%). In a global meta-analysis, Delbaz et al. [6] further confirmed that surface and drip fertigation each raised average yield by about 20%, NUE by about 26%, and irrigation water productivity by about 51%. They also found that the optimal fertilizer rate was 75% of the recommended rate, maintaining yield while cutting fertilizer input by 25%.

The limitations of fertigation, however, are equally clear: its synchronization is spatial, and the temporal dimension still relies on empirical timing and dosing. When the crop’s peak nutrient demand does not coincide with irrigation timing, mismatch persists even if water and fertilizer arrive at the root zone together in space. In a greenhouse watermelon trial, Bao et al. [56] found that an IoT-based smart drip fertigation system, which monitored soil moisture in real time and allocated fertilizer proportions by growth stage, cut irrigation water by 33%, N by 46%, P by 72%, and K by 57% over three years without reducing yield or quality. Spatial synchronization, then, is no longer the bottleneck; temporal synchronization is the key to further efficiency gains. In oilseed rape, Zheng et al. [57] showed that split fertigation (T3) raised yield and improved water productivity and nitrogen partial factor productivity relative to a single high-dose application, with soil nitrogen and water regulating yield indirectly through plant growth traits.

### 4.2. Partial-Zone and Alternate Irrigation: Trading Spatial Heterogeneity for Physiological Efficiency

Partial-zone irrigation technologies, comprising partial root-zone drying (PRD) for upland crops and alternate wetting and drying (AWD) for flooded rice, constitute the second synchronization pathway. PRD and AWD differ in physiological basis and cropping context and are discussed separately below. Both act on plant signaling rather than on delivery alone: PRD in particular improves whole-plant water-use efficiency partly by deliberately desynchronizing local water availability, which is the opposite of matching supply to demand at every point. Their contribution to synchronization is therefore indirect, through the spatial coordination of root function (PRD) and the temporal matching of redox-driven nutrient release (AWD), rather than through simple coincidence of water and nutrient supply.

PRD, in essence, divides the root system into two zones, irrigating one half at a time while the other dries, then alternating the wetted zone. The physiological elegance of the strategy is that dry-side roots keep synthesizing ABA and exporting it to the shoot through the xylem to partially close stomata and reduce transpiration, while wet-side roots maintain water uptake for basic demand [47]. In a sunflower split-root experiment, Dodd et al. [34] quantified the mechanism: alternate irrigation produced twice the peak xylem ABA concentration of fixed partial irrigation, because rewetting the dry-side roots released stored ABA into the transpiration stream as a pulse. Liu et al. [48] recently reinterpreted PRD as a spatial stress-priming mechanism, arguing that it is more than a water-saving irrigation strategy. Root-zone spatial heterogeneity triggers systemic adaptive responses, including maintained photosynthesis, osmotic adjustment, antioxidant defense, root architectural remodeling, and metabolic reprogramming, greatly broadening the biological meaning of PRD.

Sadras [58] compared PRD with conventional deficit irrigation (DI) in a meta-analysis of field performance: at equal water supply, PRD’s irrigation water productivity (IWP) was only 5% higher than DI’s (*p* = 0.007), and in 80% of comparisons the two differed by within ±20%. This invites reflection, because under field conditions PRD’s relative advantage is less pronounced than under controlled conditions, partly because field-scale soil heterogeneity already generates root-zone water gradients similar to PRD. Moreover, the PRD effect depends strongly on alternation frequency [59]: too short a cycle, at 3 days, fails to produce significant ABA accumulation, while too long a cycle, at 12 days, over-dries the dry-side roots; the optimal cycle depends on soil texture and crop species.

AWD in rice deserves separate attention because it operates in a fundamentally different soil–plant context from PRD. Unlike PRD, AWD exploits the flooding–drainage cycle of paddy fields, and periodic changes in soil redox state promote nitrogen mineralization, reduce methane emissions, and modulate cytokinin signaling [13]. In synchronization terms, AWD couples the timing of redox-driven nitrogen mineralization and availability to the crop’s uptake rhythm, making it primarily a temporal, rate-oriented mechanism, whereas PRD is primarily spatial. Its effect, however, depends heavily on water table depth and soil type. In well-drained sandy soils, frequent wet–dry cycling may increase ammonia volatilization and partly offset the water savings. Chen et al. [60] showed that adding biochar to water-saving paddy fields can effectively reduce nitrogen leaching and ammonia volatilization, offering a mitigation pathway for the environmental side effects of AWD.

More recently, in cotton, Luo et al. [61] revealed the molecular basis of hydraulic compensation in wet-side roots under PRD: JA promoted the accumulation of K+ and up-regulated aquaporin genes (GhPIP1;5, GhPIP2;8), markedly enhancing the hydraulic conductivity of wet-side roots. This JA–PIP signaling pathway underlies PRD’s enhancing-inflow effect. In the same species, Ma et al. [62] found that under PRD, the accumulation of several leaf hormones (ABA, SA, IAA, JA, JA-Ile, and cis-OPDA) was determined mainly by irrigation amount, that is, water deficit, rather than by irrigation position (PRD versus DI). The dynamics of ABA, however, were the only factor that explained the transient stomatal closure and subsequent reopening under 100% ET PRD, indicating a unique and irreplaceable role for ABA in transducing the spatial-heterogeneity signal.

### 4.3. Sensor- and Model-Driven Real-Time Dynamic Control

The third synchronization pathway centers on the deep integration of sensors, IoT, and process models, shifting the paradigm from fixed timing and dosing to on-demand, real-time control. Its key advance is to turn water–fertilizer decisions from preset schedules into real-time data-driven closed-loop optimization. This pathway also provides the setting for variable-rate irrigation and variable-rate fertilization (VRI/VRF), which are discussed in detail below.

At the sensing layer, combined deployment of soil moisture sensors (FDR/TDR), soil electrical conductivity sensors for estimating nutrient concentration, canopy-temperature sensors (thermal infrared), and hyperspectral or multispectral remote sensors enables multi-dimensional real-time monitoring of root-zone water and nutrient status and crop water stress [63]. In a review, Virnodkar et al. [63] noted that combining remote sensing with machine learning markedly improves the accuracy of crop water-stress assessment, but that current applications still lean toward post hoc evaluation rather than a real-time irrigation decision loop. Zhou et al. [64] reviewed progress in infrared thermography for assessing crop water stress in precision agriculture, arguing that deep-learning algorithms, especially convolutional neural networks (CNNs), have great potential for segmenting canopy-temperature images and grading stress. In a variable-rate center-pivot irrigation system, El-Naggar et al. [49] compared sensor-based real-time scheduling with scheduling based on the FAO56 soil water balance model and found that the sensor scheme saved 27–45% of water while maintaining yield.

At the decision layer, process models bridge sensor observations and precise decisions. HYDRUS-2D/3D can simulate the two- and three-dimensional spatiotemporal transport of water, heat, and nitrogen in the root zone, providing a physical basis for evaluating the root-zone effects of different irrigation and fertilization strategies [65]. Combining field trials with HYDRUS-3D simulation, Ding et al. [65] showed that a moderate water–nitrogen treatment (W2N2) markedly improved soil water conditions in the 0–60 cm profile and enhanced nitrogen retention, raising yield by 6.76%, WUE by 36.02%, and nitrogen partial factor productivity by 74.12% relative to the conventional control. In field trials in the Hetao Irrigation District, Li et al. [66] further confirmed that optimized water–nitrogen management raises maize productivity by strengthening coordination across the soil, root, and shoot levels, the core being root–canopy coordination: root growth, photosynthetic traits, and biomass all peaked under the I3N3 treatment (85% irrigation plus 240 kg N ha^−1^).

At the optimization layer, machine-learning algorithms are being brought into the search for water–fertilizer decisions. Baja et al. [67] used a constrained reinforcement-learning framework with a crop-growth model to explore optimal nitrogen strategies under different soil conditions, finding that the RL agent approached the optimal strategy on certain soil types but that generalization across soil textures remained the key challenge. The FDR-HY2D method of Tang et al. [50] assimilated real-time FDR sensor data into a HYDRUS-2D model to digitally reconstruct the two-dimensional 0–60 cm water distribution in the strawberry root zone, markedly outperforming SIMDualKc and AquaCrop in simulation accuracy. This data-assimilation approach bridges the information gap between discrete point observations and continuous spatial fields and is a clear success of sensor–model coupling. Cheng et al. [68] developed a real-time soil-moisture-sensor-based precision furrow irrigation system in Korea that raised winter wheat yield and WUE by 20–27% and 10–22%, respectively, over rainfed treatment.

The core bottlenecks of third-generation technology are cost, maintainability, and model generalization. A high-quality soil moisture and nutrient sensor network is expensive to deploy, with a single FDR sensor costing about US$100–300; sensor calibration is sensitive to soil salinity and temperature, and process models require extensive localized experimental data for parameterization across different soil and climate combinations [69]. As a result, third-generation technology is currently used mainly for high-value crops such as greenhouse vegetables and fruit trees and in research demonstrations, and its scale-up to field grain crops still faces economic feasibility constraints. In a sweet potato field trial at Cilembu, Indonesia, Inayah et al. [70] verified that an IoT-based smart drip fertigation system raised fertilizer-use efficiency by about 50%. Dou et al. [71] developed a vehicle-mounted real-time soil EC sensor combined with a rear variable-rate fertilizer applicator, achieving an average control error of 7.89% at a 5 m × 4 m work grid and 5 km/h forward speed, an engineering prototype for a low-cost online sensing and real-time fertilization scheme.

Variable-rate irrigation and fertilization (VRI and VRF) are the site-specific, spatial implementation of the third pathway: instead of a uniform prescription across a field, they modulate water and fertilizer inputs according to mapped or sensed variation in soil and crop status, so that the spatial dimension of synchronization is realized at sub-field resolution. The keyword term introduced at the outset is developed here. In practice, most VRI/VRF systems delineate management zones from soil apparent electrical conductivity (ECa), topography, and historical yield maps, and then assign irrigation or fertilization rates that reflect the water-holding capacity and nutrient status of each zone [72]. In a center-pivot system on the North China Plain, Zhang et al. [53] used UAV data to drive dynamic variable-rate fertigation, saving 31% of water (irrigation water productivity) while raising yield by 13% relative to uniform-rate irrigation. In a two-year field study in Mississippi, Sui and Yan [73] delineated VRI management zones based on soil ECa and found that VRI used 25% less irrigation water and produced 2.8% (soybean) and 0.8% (corn) more yield than uniform-rate irrigation, with irrigation water productivity gains of 31% and 27%, respectively. El-Naggar and Hedley [72] evaluated a commercial VRI decision-support system for maize and sweet corn across two contrasting soil zones, demonstrating its utility for prescribing spatially variable irrigation; they noted that accurate local climate data are essential for reliable scheduling. At the fertilizer end, Dou et al. [69] developed a vehicle-mounted real-time soil EC sensor coupled to a rear variable-rate fertilizer applicator, achieving an average control error of 7.89% at a 5 m × 4 m work grid and 5 km/h forward speed. These studies converge on the conclusion that VRI/VRF act directly on spatial synchronization, with the benefit depending on the resolution of the underlying soil and crop maps, on sensor accuracy, and on the machinery’s ability to execute the prescription. Broader reviews confirm that VRI and VRF remain underutilized: defining management zones and writing effective prescriptions across diverse crops and climates are unresolved challenges [74], and the wider adoption of VRF as a pillar of precision farming still faces barriers of cost, expertise, and infrastructure [75].

### 4.4. Controlled-Release Fertilizer: Matching Supply Rate to Demand Rhythm

Controlled-release fertilizer (CRF) represents the fourth synchronization pathway, which addresses synchronization from the supply side. Unlike the water-delivery pathways, which synchronize by regulating irrigation timing or space, CRF changes the kinetics of nutrient release by physicochemical means, with the aim that the release-rate curve approximates the crop’s uptake curve. Slow or controlled release does not by itself guarantee synchronization: release kinetics depend on coating properties, temperature, soil moisture, microbial activity, fertilizer placement, and other environmental conditions, while crop demand changes with phenology and stress, so synchronization occurs only when release kinetics sufficiently match crop demand under the relevant environment.

Polymer-coated urea (PCU) and sulfur-coated urea (SCU) are the two most mature controlled-release nitrogen products. Their design principle is that the thickness and porosity of the coating determine the rate at which water enters to dissolve the urea core, and thus the release rate of the urea solution. Under ideal conditions, nutrient release follows a sigmoidal curve, with an initial slow phase, a linear rapid phase, and a final decay phase, which matches the crop’s sigmoidal logistic nitrogen-uptake curve reasonably well [52].

In winter wheat, Ma et al. [52] compared one-time basal application with two split applications (pre-sowing and regreening) of PCU and SCU and found that two split applications of controlled-release nitrogen fertilizer matched the crop’s staged nitrogen demand markedly better than one-time basal application. The split treatment kept post-anthesis soil inorganic nitrogen at a higher level, satisfied the second nitrogen demand peak, and significantly increased post-anthesis dry-matter accumulation, raising yield by 9.96–15.11% and NUE by 6.71–10.33% relative to split urea topdressing. This reveals a key insight: even a carefully designed release curve may deviate from the actual field demand curve because of variation in seasonal weather, soil temperature, and moisture, so controlled release is not the same as intelligent release.

At the national scale, a meta-analysis by Zhang et al. [53] showed that controlled-release urea (CRU) is generally a good substitute for split urea topdressing; in oilseed rape, Xiao et al. [76] confirmed that CRU at 240 kg N ha^−1^ raised grain nitrogen accumulation by 115.7–149.3% over one-time urea application. Nationally, CRU raised yield by 10.08% and NUE by 47.55%, whereas split topdressing raised them by 8.11% and 45.21%. Under certain conditions, however, CRU substitutes poorly: low soil available nitrogen, below 50 mg kg^−1^; low nitrogen rate, below 150 kg ha^−1^; and wheat as the crop. This is consistent with the recent finding of Ma et al. [52] that CRF improves the nitrogen supply–demand synchronization index (SDI) from 0.61 for conventional fertilizer to 0.77–0.86, shifting the regulation of yield formation from intensity-driven nitrogen supply to a synchronization-driven mode of nutrient redistribution.

In terms of application innovation, Ahmad et al. [77] combined controlled-release blended fertilizer with subsurface application in paddy soil and, by activating key nitrogen-metabolism enzymes (NR, NiR, GS, GOGAT) and up-regulating nitrogen transporter genes (NRT2.1, AMT1, GS1, GOGAT1), reduced ammonia volatilization by 37% and increased nitrogen uptake by 39%. In relay-intercropped maize, Feng et al. [78] found that slow-release fertilizer reshaped root traits, elongating laterals by 19–21% and increasing root biomass in the 18–42 cm layer, and raised post-anthesis nitrogen assimilation and nitrogen agronomic efficiency by 40.9%. Together, these studies show that the benefit of CRF lies not only in release–uptake timing but also in the linked effects of root architectural remodeling and nitrogen-metabolism activation. From a materials-science angle, Sim et al. [79] reviewed recent advances in biochar-based slow- and controlled-release fertilizer, including biochar’s nutrient-loading capacity, modification methods, and release mechanisms, opening technical possibilities for combining waste valorization with precise nutrient management.

CRF offers a technical pathway to water–fertilizer synchronization distinct from regulating irrigation. The irrigation route targets precise delivery on the demand side, while the controlled-release route targets intelligent release on the supply side, and the two are complementary rather than substitutive. The current challenge is to combine their strengths. Using partly controlled-release fertilizer in a drip system, for instance, reduces the number of topdressings, and pairing it with soil moisture sensors to regulate irrigation in real time may improve coordination among nutrient release, root-zone delivery, and crop uptake, rather than delivering demonstrated end-to-end spatiotemporal coordination. The potential value of integrating management components is also supported by field evidence showing that coordinated water, nutrient, and soil-surface management can improve crop yield efficiency, although the magnitude of these benefits depends on the specific production environment [80].

**Figure 3:**
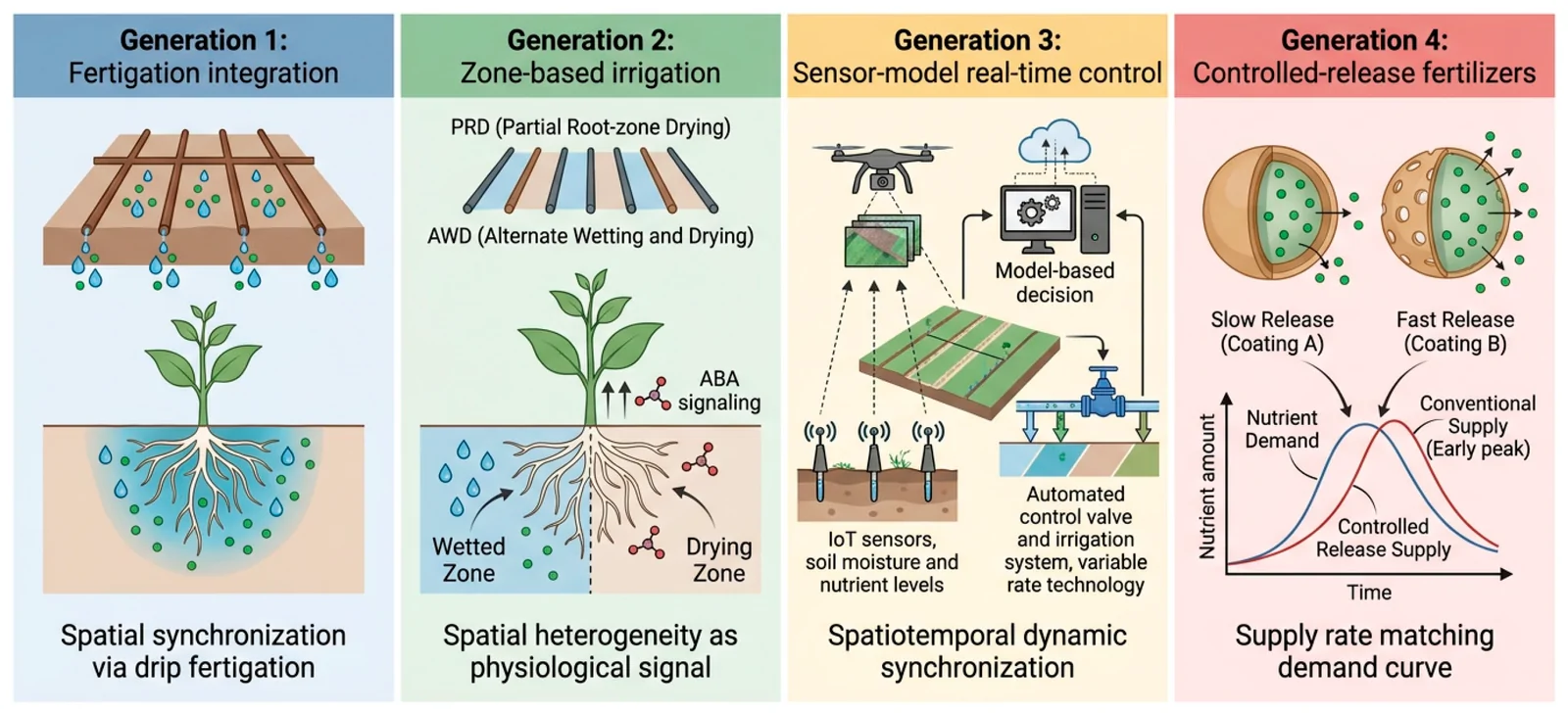
The four complementary pathways of water–fertilizer regulation, from simultaneous application to synchronized supply (an organizational framework, not a chronological sequence).

As Figure 3 shows, the four pathways do not form a linear replacement but a progressive layering of regulatory capability, moving from spatial synchronization to temporal, then to spatiotemporally dynamic, then to rate synchronization. They are complementary and partly independent: in a given application, the low-cost first pathway, drip fertigation, may be combined with the fourth, CRF, to strike the best balance between cost and precision.

## 5. Divergence in the Effects of Synchronized Supply: Why No Universal Optimum Exists

From fertigation to controlled-release fertilizer, the four pathways generally show yield and efficiency gains under controlled conditions, but the magnitude varies widely with crop, environment, experimental design, and comparator treatment. Yet when these optimal schemes are extended to different soils, crop cultivars, climate years, and spatial scales, the consistency of the effect drops sharply. This section analyzes the divergence of synchronized-supply effects and its mechanistic basis, along four dimensions: soil physicochemical and hydraulic properties, genotype, climate year, and spatial scale.

### 5.1. How Soil Physicochemical and Hydraulic Properties Reconstruct Water and Nutrient Transport Pathways

Soil texture is a readily characterized but not the sole driver of synchronized-supply effects; soil structure, bulk density, organic matter, mineralogy, pH, cation-exchange capacity, hydraulic conductivity, water-retention characteristics, rooting depth, and preferential flow can modify water and nutrient dynamics to a comparable degree. Texture is used here as a first-order proxy, while the broader category of soil physicochemical and hydraulic properties is the operative dimension, acting by changing the residence time and spatial distribution of water and nutrients in the root zone. In sandy soils, high saturated hydraulic conductivity lets irrigation water infiltrate rapidly through the root zone into depth, carrying nitrate nitrogen with the wetting front below the root layer; in clayey soils, low conductivity and high water-holding capacity prolong the residence time of water and nutrients in the root zone but may also reduce aeration and thereby limit root respiration and active nutrient uptake [81].

Using 40 years of meteorological data and 5000 soil samples with the BDSSAT model, Chen et al. [81] compared soil water and nitrogen cycling and maize yield under biodegradable mulch (BM) across five soil textures and rainfall zones, finding that the water–nitrogen balance difference between BM and plastic mulch (PM) was smallest in loam areas and largest in sandy loam and sand. In the loam and high-rainfall combination, BM even outperformed PM, contrary to the conventional belief that plastic mulch suits arid sandy soils.

Tougma et al. [82] used a process model to assess the economic performance and nitrogen loss of 15 rotations on loam and sand, finding that loam achieved the highest economic return and lowest nitrogen loss under all rotations, and that soil organic carbon increased more on loam than on sand but with greater interannual variation. This duality of return and risk means that optimizing synchronized supply for soil texture cannot rest on average effects alone; interannual stability matters, which is critical for the adoption decisions of risk-averse farmers.

Using X-ray CT, Rogers et al. [83] quantified the three-dimensional RSA of eight rice cultivars in five substrate textures, finding that genotype × substrate interaction dominated RSA variation and that certain cultivars, such as Azucena, showed a stable, low-plasticity architecture across all substrates. This bears directly on the design of synchronized-supply technology: in fields with high textural variability, choosing a cultivar with low RSA plasticity, that is, one insensitive to soil texture, may be more practical and cost-effective than finely adjusting irrigation and fertilization for each field. Recent work from a xylem-anatomy angle has shown how soil texture regulates drought resistance by affecting root hydraulic structure: Strock et al. [84] found that genotypic variation in maize metaxylem vessel element length (MVEL) was closely related to root axial hydraulic conductivity, rooting depth, deep-water use, and yield under drought, a stretch phenotype whose explanatory variables ultimately interact with the hydraulic properties of soil texture.

### 5.2. Genotypic Differences: Root Traits as Response Modulators

Soil physicochemical and hydraulic properties set the physical boundaries of water and nutrient movement, while crop genotype determines how roots respond to and use those resources within those boundaries. It is more informative to treat genotype not as a categorical source of variation but as a set of measurable traits that modulate the response: rooting depth and distribution, root hydraulic conductance, stomatal sensitivity to ABA and vapor pressure deficit, aquaporin regulation, nutrient-transporter responses, phenology, and nutrient-demand dynamics. These traits connect the physiological mechanisms of Section 3 to the technological pathways of Section 4. Genetic variation in root architecture, aquaporin expression, and ABA sensitivity makes the response to synchronized supply differ markedly in magnitude.

Jabborova et al. [85] found that co-application of biochar and arbuscular mycorrhizal fungi significantly promoted soybean root growth and soil enzyme activity under drought. Using O-isotope tracing, Corneo et al. [86] revealed contrasting water-uptake strategies of four wheat genotypes in a clay Vertosol and a sandy Kandosol: in sand, genotype 249 tended to take up water from shallower drying layers, whereas F6 and Suntop relied more on deep water; in clay, Suntop and F6 had greater root branching intensity in the topsoil (0–10 cm). More importantly, genotypes with greater mean water-uptake depth yielded more in sand, indicating that a deep-root strategy is advantageous in sand while a shallow-branching strategy is better in clay.

Vandeleur et al. [36] showed in grape how differences in aquaporin expression determine cultivar water strategies. The isohydric cultivar Grenache down-regulated root AQP expression and lowered root hydraulic conductance under drought, reducing transpiration and maintaining a relatively stable leaf water potential, whereas the anisohydric cultivar Chardonnay maintained or even increased AQP expression, sustained transpiration under high VPD, and showed a marked drop in leaf water potential. In cotton, Wang et al. [87] further confirmed the decisive role of root hydraulic architecture, integrating morphological and hydraulic traits, in cultivar drought resistance: drought-resistant cultivars coordinated morphology and hydraulics by increasing lateral-root length and number, reducing lateral-root tip angle, lowering the root width-to-depth ratio, and optimizing xylem vessel diameter and cortical cell layers, so as to maintain higher root hydraulic conductance under drought. For synchronized supply, this implies that isohydric cultivars may have a narrower response window, having their own strong water-deficit avoidance, whereas anisohydric cultivars may gain more from real-time water and fertilizer regulation.

For nitrogen efficiency, significant genotypic variation in NUE has been found in both wheat [88] and eggplant [89], and nitrogen utilization efficiency (NUtE), the grain yield per unit of nitrogen taken up, is the main component driving variation in total NUE. Gaju et al. [88] found that, under low nitrogen, a later onset of post-anthesis senescence explained 32–70% of the genotypic variation in NUtE. The mechanism is that delayed senescence lengthens the photosynthetic period and raises post-anthesis carbon and nitrogen assimilation, giving the plant more time to remobilize assimilated nitrogen to the grain.

Taken together, genotypic variation has far-reaching implications for synchronized-supply technology. After an optimal water–fertilizer scheme is obtained for a given soil texture and climate, changing cultivar may require re-optimizing or even redesigning the scheme. Future water and fertilizer management should move from a general scheme with cultivar tuning toward a cultivar–scheme matching framework. Field evidence in common bean likewise shows that crop responses depend jointly on irrigation management, nitrogen fertilization, and cultivar, supporting the view that genotype is an integral component of water–nutrient management rather than a separate factor [90]. In cotton, Guo et al. [91] used a rain-shelter water-control system to compare RSA changes among cultivars of differing drought resistance, finding that drought-resistant cultivars optimized RSA by increasing mean lateral-root length by 34% and maximum rooting depth by 15%, and that seed-cotton yield correlated positively and linearly with specific root length, mean lateral-root length, and maximum rooting depth, and negatively with root tissue density and lateral-root angle, a quantitative relation that offers actionable breeding indicators for cultivar–scheme matching.

### 5.3. Interaction of Interannual Climate Variability with Spatial-Scale Effects

The effect of synchronized supply depends not only on soil and genotype but also strongly on interannual climate variability and research scale. In dry years, the yield and efficiency gains of synchronized supply are usually much larger than in wet years, because natural rainfall in wet years already meets part of the crop water demand and the marginal contribution of irrigation control declines. Fan et al. [2] confirmed this in a global meta-analysis: in arid and semi-arid environments, crops were more sensitive to reduced water–nitrogen coupling than in sub-humid environments (potato > maize > wheat) [2], and for the same reduction in water and nitrogen input, potato suffered the greatest yield loss and wheat the least.

Scale effects are another often-overlooked source of divergence. A fertigation scheme validated as optimal in pot or plot trials often loses effect when extended to field or regional scale, because soil spatial heterogeneity amplifies the variation. Using 30 years of simulation, Hernández-Ochoa et al. [92] quantified spatiotemporal yield variation at the within-field daily scale, finding that for most crops, spatial variation, at 17.3–45.8%, exceeded temporal variation, at 10.4–36.8%, and that maize and sunflower showed a significant interaction between soil available water capacity and seasonal rainfall. This means that even within a single field, the optimal water–fertilizer scheme differs among soil micro-zones.

The core effect of a scale jump is not only attenuation but possible reversal of effect direction. In a three-season maize trial at two sites in Kenya, Enock et al. [93] found that among 54 water × nitrogen-source treatment combinations, 94% were additive and 6% antagonistic, with no synergistic effect. This challenges the mainstream narrative of water–nitrogen synergy: in resource-limited dryland systems, the interaction between water and nitrogen may be mainly additive rather than synergistic, so stepwise adoption of low-cost soil water conservation measures may be more economical than investing in expensive synchronized-supply equipment. The same authors [93] further suggested adopting measures stepwise, starting with low upfront cost and advancing to costlier options after capital has accumulated, rather than investing in a full synchronized-supply package at once. More recent field evidence reinforces that crop responses to combined water and nutrient management depend strongly on the production environment and resource regime, so a yield gain under joint management should not automatically be read as physiological synergy between water and nutrient supply [94].

Sun et al. [95] proposed a UAV hyperspectral method for water–nitrogen coupling decisions, using an SPA-RF model to estimate leaf water content (R2 = 0.83) and a VIs-RF model to estimate chlorophyll content (R2 = 0.65), then linking these through a conversion model to soil water content and leaf nitrogen content and achieving on-demand dynamic adjustment of water and nitrogen application. The advantage of such real-time remote-sensing feedback is that it can partly offset the uncertainty from seasonal climate variability. At present, however, the soil water content conversion model reaches only R2 = 0.79, and prediction errors remain large under extreme drought or extreme wetness. Yang et al. [96] used a low-cost thermal infrared camera with an EfficientNet-B0 model to classify watermelon water stress accurately, at 0.99 accuracy, showing the potential of lightweight AI models for field-deployable irrigation systems. From a macro environmental angle, an LCA by Medel-Jiménez et al. [97] showed that variable-rate nitrogen application guided by optical sensors for winter wheat reduced the carbon footprint by 8.6% relative to uniform application, while the contribution of ICT components such as sensors, internet, and computers was below 1% in all environmental impact categories. The environmental cost increment of precision fertilization is therefore nearly negligible, and fertilizer production and field emissions remain the main sources of impact. In a 12-year trial in Northern France, Belleville et al. [98] confirmed that the C:N ratio of crop residues, rather than the amount applied, was the key factor regulating cumulative emissions.

Across the four dimensions, the essence of divergence in synchronized-supply effects is the four-way interaction of soil texture, genotype, climate year, and spatial scale: a scheme optimal along one dimension may degrade to sub-optimal or even ineffective under other conditions. The resource requirements associated with exploitable yield are likewise crop- and environment-dependent; analyses in tropical soybean show that attainable yield intensification must be considered in relation to both water and nutrient demand rather than either resource in isolation [99]. This leads to the core conclusion of this section. Current evidence does not support a universally optimal water–nutrient management scheme across contrasting soils, genotypes, climates, and production scales, and water and fertilizer management must move from seeking the optimal scheme to building context-specific management capacity, the ability, for a given combination of soil, genotype, and climate, to select or combine the most suitable pathway.

As Table 3 shows, a marked gap remains between the divergence mechanisms along each dimension and current response strategies. Current strategies mostly average out heterogeneity through texture-zoned management and multi-year mean schemes, whereas future directions point to heterogeneous precision management through smart zoning and adaptive control. This shift from homogenization to heterogenization is the essence of context-specific management.

## 6. Research Gaps and Future Directions

Although research on synchronized water–fertilizer regulation has advanced considerably over the past two decades, three interrelated bottlenecks persist across the chain from mechanistic understanding to technological innovation to field application. Together, they form a knowledge triangle gap that holds the field back: weak in situ observation at the root–soil interface, the absence of coupled multi-process models, and scarce field validation data.

### 6.1. Technical Bottlenecks in In Situ Dynamic Observation of the Root–Soil Interface

The root–soil interface is the core physical space of synchronized water–fertilizer regulation, and also the weakest link in current observational capability. The main existing techniques each have a focus, but all have notable limitations. Neutron imaging can visualize root-zone water distribution and root water-uptake dynamics, but its spatial resolution is limited, at about 50–100 μm, and it requires a nuclear reactor or accelerator as a neutron source, making it costly with limited throughput [100]. X-ray CT offers higher spatial resolution, at 10–50 μm, and can finely reconstruct three-dimensional root architecture, but it struggles to distinguish water from nutrient concentration distributions simultaneously [83]. Planar optodes and zymography can quantify the two-dimensional distribution of rhizosphere pH, oxygen, and enzyme activity at high spatial resolution, at 50–100 μm [37], but their measurement range is confined to the two-dimensional surface of transparent root boxes and is hard to extend to three-dimensional field conditions.

Using hydrogen and oxygen stable isotopes with a MixSIAR Bayesian mixing model, Liu et al. [101,102] revealed the profile distribution of wheat root water uptake under different nitrogen rates and the quadratic relation between soil evaporation loss and the ratio of root water uptake, offering a non-destructive way to infer root water-uptake function indirectly at field scale. The temporal resolution of the isotope method is limited by the sampling interval, usually 7–14 days; however, it cannot capture daily or hourly dynamics. The most pressing technical need is a platform that can image water, nutrients, and roots simultaneously, non-destructively, and in three dimensions in situ. One possible direction is multimodal fusion of X-ray CT and neutron imaging: using the high spatial resolution of X-ray CT to reconstruct three-dimensional root structure, the high hydrogen sensitivity of neutron imaging to quantify root-zone water distribution, and fluorescence tracing to label specific nutrient elements such as N and P. The core challenge of multimodal fusion, however, is not the hardware but how to balance acquisition timing, spatial resolution, and radiation dose across imaging modes, and how to develop registration and reconstruction algorithms that handle heterogeneous data. This multimodal approach is a prospective research direction rather than an immediately deployable solution: at present, no single platform images water, nutrients, and roots simultaneously and non-destructively in situ under field conditions.

### 6.2. The Translation Gap from Mechanistic Understanding to Field Validation

The attenuation of fine-control schemes obtained under controlled conditions when moved to the field, the mechanism-to-field translation gap, is a challenge shared across disciplines. In synchronized water–fertilizer regulation, attenuation has three main sources. The first is amplified soil spatial heterogeneity: the uniformly packed soil substrate of controlled experiments cannot represent the sharp meter- or even decimeter-scale changes in soil texture under field conditions [103]. The second is the uncontrollability of climate: irrigation and fertilization schedules preset in controlled experiments are often disrupted in the field by rainfall events, extreme temperatures, and changes in wind speed. The third is the decline of management precision: manual precise weighing and timed operation in experimental plots are hard to replicate in large-scale mechanized operations.

Using ECa (apparent electrical conductivity) scanning and the Sirius crop model in a long-term UK trial, Clarke et al. [103] quantified the spatial variation in NUE within a 3 ha field. At 220 kg N ha^−1^, the five-year average yield in low-ECa zones was 9.0 t ha^−1^ with grain protein 11.6%, versus 10.7 t ha^−1^ and 11.0% in high-ECa zones. Model simulation further showed that the optimal nitrogen rate in low-ECa zones averaged 12 kg N ha^−1^ lower, and in more than a quarter of years the optimal rate was below 200 kg N ha^−1^. This indicates that even within one field, the optimal water–fertilizer scheme differs markedly among zones because of small differences in soil properties, so one can expect the divergence to widen further as the scale extends from a single field to a region.

Bridging the translation gap requires a four-tier validation system spanning pot, plot, farm, and region. Pot trials serve mechanistic analysis and initial scheme screening; plot trials serve scheme optimization and multi-environment validation; farm trials serve feasibility and economic assessment; and regional-scale validation serves policy support and the design of extension programs. At each scale transition, process models should be used for scaling and uncertainty quantification. HYDRUS, for example, can simulate root-zone water and nitrogen transport; DSSAT and APSIM can simulate crop-growth responses, and Monte Carlo methods can estimate how uncertainty in model parameters affects the optimal scheme. An LCA by Munnaf et al. [104] showed that variable-rate nitrogen fertilization (VRNF) delivered better environmental sustainability under Belgian than Spanish conditions, and that nitrogen allocation based on the King’s Approach (KA) was more productive than the Robin Hood (RH) approach, indicating that the direction of the effect of a precision fertilization scheme depends both on the geoclimatic context and on the design logic of the decision algorithm, which reinforces the need for multi-environment validation.

### 6.3. An Intelligent Closed-Loop System Integrating Sensing, AI, and Decision-Making

Cost, power consumption, and maintenance are the current scale-up bottlenecks of sensor–IoT systems, but the technology trend is moving favorably. Monolithic MEMS sensors and multi-parameter integrated sensing chips are compressing the cost per monitoring point from hundreds of dollars to tens of dollars [105]. The dual-sensor Stem-FIT and Soil-FIT platforms developed by Hossain et al. [105] can simultaneously and in real time monitor nitrate, ammonium, pH, and plant hormones (SA, IAA, MeJA, ET) in the plant stem and the corresponding parameters in soil, and, combined with an LSTM model, they achieved short-term prediction of future nutrient–hormone fluctuations, up to R2 = 0.86, demonstrating the feasibility of multi-parameter sensing plus AI prediction. A systematic review by Sharma et al. [106] noted that the fusion of plant biosensors with machine learning is becoming a new frontier in precision agriculture but faces challenges such as scarce labeled data and difficult model transfer across crops. Edge AI chips, such as Google Coral TPU and NVIDIA Jetson Nano, now consume only 3–10 W, allowing solar-plus-battery-powered standalone field nodes to run for months without power replacement. The maturation of low-power wide-area networks (LoRaWAN) lets hundreds of field nodes aggregate data to a single gateway for tens of dollars a year in communication cost [51]. These hardware advances are moving a low-cost sensor, edge-AI, and digital-twin closed loop from proof of concept to engineering feasibility.

At the algorithmic level, reinforcement learning (RL) and deep learning offer new tools for the dynamic optimization of water and fertilizer decisions. The constrained RL framework of Baja et al. [67] demonstrated the near-optimality of a nitrogen-strategy optimization based on crop-growth-model simulation for certain soil types but also revealed insufficient generalization across soil types. The VGG16-EF-CNN multimodal fusion model of Elsherbiny et al. [107] reached 97.9% accuracy in predicting winter wheat irrigation frequency, indicating considerable potential for deep-learning methods that fuse visual images and environmental sensor data in decision support. The current bottleneck of these AI methods, however, is not the model architecture but the scarcity of labeled training data: obtaining high-quality labeled data covering many soil, weather, and cultivar combinations requires years of systematic field experiments across sites.

Digital-twin technology is one of the ultimate technical forms of future water and fertilizer management. Its core idea is to build a continuously updated virtual replica of each field, which keeps receiving water, nutrient, weather, and crop-growth data from field sensors and, through a hybrid process-model and AI approach, simulates the crop state and resource-use efficiency over the coming days, then uses optimization algorithms to search for the optimal irrigation and fertilization scheme and execute it automatically [108]. This architecture has seen initial application in protected agriculture; a digital irrigation system for greenhouse strawberry, for example, has put the digital-twin concept into practice [50]. Extending it to field crops still faces substantial challenges in sensor density, communication reliability, and real-time model correction. In Arabidopsis, Cáceres et al. [109] confirmed that root hydraulic conductance can actively respond to changes in shoot transpiration demand, a root–shoot hydraulic dialog that provides a key biological constraint for parameterizing the root module of a digital twin. A review by Dong et al. [110] noted that although UAV remote sensing provides a flexible and efficient platform for monitoring crop water stress, achieving a truly automated loop from stress diagnosis to irrigation decision execution still requires solving key bottlenecks such as data-assimilation algorithms and non-stomatal limitation effects. In smallholder sugarcane fields in South Africa, Yacoob et al. [111] fused satellite and UAV data and used machine learning to predict NDWI as a water-stress index (R2 = 0.95), providing a low-cost remote-sensing diagnostic scheme for resource-limited settings.

Predictive performance does not by itself guarantee biological interpretability, transferability across environments, or agronomic robustness. AI models are constrained by their training domain and by sensor uncertainty, missing data, and model drift over time, and their outputs remain conditional on genotype × environment interactions. AI is therefore best presented as a decision-support layer that informs human management choices, rather than as a physiological solution to synchronization.

**Figure 4:**
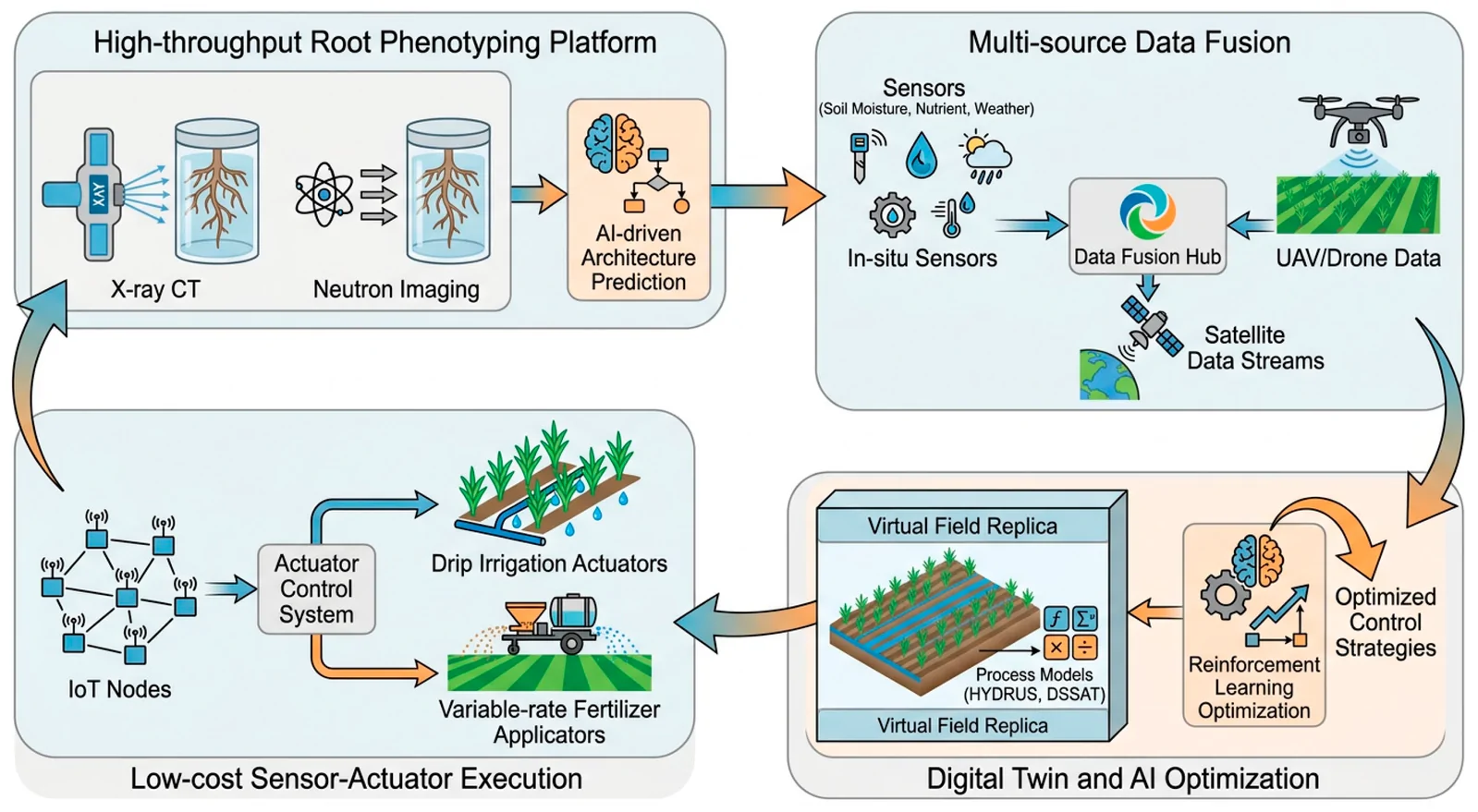
Future pathway for synchronized water–fertilizer regulation: an intelligent closed-loop system architecture.

As Figure 4 shows, future synchronized water–fertilizer regulation will move from the current linear mode of preset schemes, manual execution, and post hoc evaluation to a continuous closed loop of sensing, prediction, decision, execution, and feedback. The core driver of this transition is the convergence of falling hardware costs and maturing AI algorithms, which may facilitate wider adoption of precision water and fertilizer management. Whether this potential is realized will depend on farm scale, infrastructure, technical expertise, maintenance, data connectivity, economics, and the characteristics of regional production systems, so the transition should be read as prospective rather than assured.

### 6.4. Environmental Trade-Offs of Synchronized Supply

Higher yield, water-use efficiency, or nitrogen-use efficiency does not by itself imply better environmental performance, so the review’s environmental dimension warrants explicit treatment. Nitrate leaching remains a risk where irrigation increases drainage below the root zone, especially in sandy soils and under over-irrigation. Nitrous oxide emissions depend on soil moisture, temperature, nitrogen form, and the reference management treatment. Ammonia volatilization is sensitive to placement, rate, and surface moisture. Water-saving regimes also carry trade-offs, because frequent wet–dry cycling can alter gaseous nitrogen losses, while controlled-release fertilizers raise questions about the fate of coating residues. Rebound effects may further erode field-level efficiency gains when higher water or nutrient productivity encourages intensification or area expansion.

The quantitative results below are study-specific observations, not universal performance ranges for synchronized management. In the sandy-soil and excessive-irrigation context examined in [112], 32–61% of applied N was reported to move below the root zone. In the coarse-sandy-soil oilseed-rape and spring-barley comparison in [113], irrigation synchronized with crop demand was associated with leaching reductions from 127 to 85 kg N ha^−1^ and from 72 to 42 kg N ha^−1^, respectively, relative to the study’s comparison treatments. The meta-analysis in [114], based on 485 field observations, reported lower N_2_O emissions under drip than under furrow or sprinkler irrigation and a mean fertilizer-induced emission factor of 0.35%; these estimates reflect the included experiments and their baselines. Likewise, the NH_3_ and N_2_O changes reported in [115], the net energy savings estimated for the groundwater-pumping comparison in [116], the polymer-residue mass and incubation losses reported in [117], and the China-specific irrigation rebound estimate in [118] each depend on their respective management systems, locations, time horizons, and calculation bases. They therefore identify environmental trade-offs that require site-specific assessment, rather than characterizing synchronized water–nutrient management universally.

## 7. Conclusions

The proposal of synchronized water–fertilizer supply and the growth of related research reflect a shift in crop water and fertilizer management, from empirical simultaneous application toward mechanistically guided synchronization. Using a four-part, progressively nested framework, covering the cost of supply–demand mismatch, sensing and response mechanisms, complementary technological pathways, and sources of effect divergence, this review has traced the basis and progress of that shift. The three-tier sensing–response framework and the four-pathway organization used throughout this review are the authors’ integrative interpretation rather than an established canonical model.

At the level of physiological mechanism, the feasibility of synchronized water and fertilizer supply rests on the root’s three-tier sensing–response system. RSA plasticity provides spatial orientation through the differentiated signaling pathways of hydrotropism and nutrient-dependent foraging; the dual hydraulic and chemical signaling system coordinates root and shoot through complementary timescales; and rhizosphere processes contribute to the biological synchronization of heterogeneous water and nutrient distribution through dynamic feedbacks among root exudation, microbial processes, and nutrient availability. These three tiers form a layered yet coupled support network across the spatial, temporal, and biological dimensions, offering intervention targets for technology development.

At the level of technological pathway, fertigation primarily targets root-zone resource delivery and spatial co-location; partial root-zone or alternate irrigation manipulates root-zone water heterogeneity, plant signaling, or redox cycling; sensor- and model-driven control supports data-informed adjustment of water and nutrient decisions; and controlled-release fertilizer targets the timing and rate of nutrient release. These pathways address distinct mechanisms and may be combined where their functions are complementary. The relative value of any combination is context-dependent. For example, PRD plus controlled-release fertilizer and conventional drip fertigation under contrasting soil physicochemical and hydraulic properties, genotypes, and seasonal climates should be understood as illustrative management scenarios, not as empirically demonstrated rankings of specific technology combinations.

At the level of effect assessment, constrained by the four-way divergence of soil physicochemical and hydraulic properties, genotype, climate year, and spatial scale, the effect of synchronized supply is highly context-dependent when scaled up. This supports the conclusion that current evidence does not indicate a universally optimal scheme and points to a needed shift in the field’s direction: the research focus should move from whether supply can be synchronized to which synchronization scheme to choose under which context, that is, to establishing a new paradigm of context-specific management.

Taken together, the conclusions fall into four categories. It is well established that water and nutrient supply and crop demand can mismatch in time and space, that roots sense and integrate heterogeneous water and nutrient signals, and that the management technologies reviewed improve resource-use efficiency under controlled conditions and, for several, under field conditions. It remains context-dependent whether a given scheme raises yield or efficiency at a specific site, because soil properties, genotype, climate year, and scale interact. It is mechanistically plausible but incompletely validated that deliberate synchronization produces gains beyond those of independent water and nutrient management, and that reductionist physiological findings extrapolate to field-scale yield. And it requires field-scale testing to establish quantitative synchronization metrics, the magnitude of the hidden yield penalty under mismatch, and the environmental net balance of these technologies.

Looking ahead, breakthroughs in three areas will determine whether synchronized water–fertilizer regulation can move from a scientific concept to large-scale agricultural practice. First, the development of simultaneous, non-destructive, three-dimensional dynamic imaging of water, nutrients, and roots at the root–soil interface will provide the in situ observational capability that research on synchronization mechanisms most lacks. Second, a fusion system of low-cost sensors, edge AI, and digital twins is expected to turn precision water and fertilizer decisions from a high-cost research tool into a scalable production tool. Third, the establishment of a multi-environment, multi-year field validation network, systematic validation covering major crops, soil types, and climate zones, is the necessary path from mechanistic understanding to practical extension. Only by crossing these three bottlenecks can water and fertilizer management move substantially from empirical simultaneity toward evidence-based synchronization.

## Figures and Tables

**Table 2:** Four complementary technological pathways for water–fertilizer regulation, their intervention level, and the synchronization dimension each addresses.

Pathway	Paradigm	Intervention Level	Synchronization Dimension	Core Mechanism	Representative References
1	Fertigation	Resource delivery	Spatial	Concentrated root-zone supply	[6,7]
2	Partial-zone and alternate irrigation	Physiological manipulation	Spatial + temporal	ABA signaling and wet–dry cycling	[34,47,48]
3	Sensor–model real-time control	Decision and control	Spatiotemporally dynamic	Data-driven closed-loop decisions	[49,50,51]
4	Controlled-release fertilizer	Fertilizer formulation	Supply rate	Physicochemical slow release	[52,53,54]

Note: Benefit data for the first pathway come from the China meta-analysis of Li et al. [7] and the global meta-analysis of Delbaz et al. [6]; the PRD mechanism of the second pathway follows the split-root experiments of Dodd et al. [34,47] and the spatial stress-priming extension of Liu et al. [46]; the third pathway refers to the quantitative assessment by Singh and Singh [51] of a 61–64.1% WUE gain in precision fertigation; and the synchronization index (SDI) improvement for controlled-release fertilizer in the fourth pathway is drawn from Ma et al. [54].

**Table 3:** Key dimensions of divergence in synchronized-supply effects, representative evidence, and response strategies.

Dimension	Core Mechanism (Representative References)	Current Strategy	Future Direction
Soil physicochemical and hydraulic properties	Reconstructed transport pathways [81,83]	Texture-zoned management	Digital soil mapping and smart zoning
Genotype	Root response regulation [86,91]	Cultivar–scheme matching	GWAS-assisted root-trait breeding
Climate year	Fluctuating marginal benefit [2]	Multi-year mean scheme	Seasonal forecasting and adaptive control
Spatial scale	Heterogeneity dilution [92,93]	Plot-level optimized scheme	Multi-scale validation and scaling models

Note: Soil-property data come from the 40-year BDSSAT simulation of Chen et al. [81] and the X-ray CT analysis of rice RSA by Rogers et al. [83]; genotype effects follow the O-isotope tracing of Corneo et al. [86] and the quantitative drought-resistance RSA relations of Guo et al. [91]; climate-year patterns are drawn from the global meta-analysis of Fan et al. [2]; and scale-effect evidence comes from the 30-year simulation of Hernández-Ochoa et al. [92] and the Kenya field trial of Enock et al. [93].

## Data Availability

No new data were created or analyzed in this study. Data sharing is not applicable to this article.
